# αvβ3 integrin-specific exosomes engineered with cyclopeptide for targeted delivery of triptolide against malignant melanoma

**DOI:** 10.1186/s12951-022-01597-1

**Published:** 2022-08-23

**Authors:** Yongwei Gu, Yue Du, Liangdi Jiang, Xiaomeng Tang, Aixue Li, Yunan Zhao, Yitian Lang, Xiaoyan Liu, Jiyong Liu

**Affiliations:** 1 Department of Pharmacy, Fudan University Shanghai Cancer Center, Fudan University, Shanghai, 200032 China; 2 Department of Oncology, Shanghai Medical College, Fudan University, Shanghai, 200032 China; 3 Department of Pharmacy, Children’s Hospital Affiliated to Shanghai Jiao Tong University, Shanghai, 200062 China; 4 School of Pharmacy, Shanghai Jiao Tong University, Shanghai, 200240 China; 5 Department of Pharmacy, Huangpu Branch, Shanghai Ninth People‘s Hospital, Shanghai Jiao Tong University School of Medicine, Shanghai, 200011 China; 6 State Key Laboratory of Quality Research in Chinese Medicine & School of Pharmacy, Macau University of Science and Technology, SAR, Avenida Wai Long, Taipa, 999078 Macau China

**Keywords:** Exosomes, Targeted delivery, cRGD, Triptolide, Malignant melanoma

## Abstract

**Background:**

Melanoma is the most malignant skin tumor and is difficult to cure with the alternative treatments of chemotherapy, biotherapy, and immunotherapy. Our previous study showed that triptolide **(**TP) exhibited powerful tumoricidal activity against melanoma. However, the clinical potential of TP is plagued by its poor aqueous solubility, short half-life, and biotoxicity. Therefore, developing an ideal vehicle to efficiently load TP and achieving targeted delivery to melanoma is a prospective approach for making full use of its antitumor efficacy.

**Results:**

We applied exosome (Exo) derived from human umbilical cord mesenchymal stromal cells (hUCMSCs) and engineered them exogenously with a cyclic peptide, arginine-glycine-aspartate (cRGD), to encapsulate TP to establish a bionic-targeted drug delivery system (cRGD-Exo/TP), achieving synergism and toxicity reduction. The average size of cRGD-Exo/TP was 157.34 ± 6.21 nm, with a high drug loading of 10.76 ± 1.21%. The in vitro antitumor results showed that the designed Exo delivery platform could be effectively taken up by targeted cells and performed significantly in antiproliferation, anti-invasion, and proapoptotic activities in A375 cells via the caspase cascade and mitochondrial pathways and cell cycle alteration. Furthermore, the biodistribution and pharmacokinetics results demonstrated that cRGD-Exo/TP possessed superior tumor targetability and prolonged the half-life of TP. Notably, cRGD-Exo/TP significantly inhibited tumor growth and extended survival time with negligible systemic toxicity in tumor-bearing mice.

**Conclusion:**

The results indicated that the functionalized Exo platform provides a promising strategy for targeted therapy of malignant melanoma.

**Graphical Abstract:**

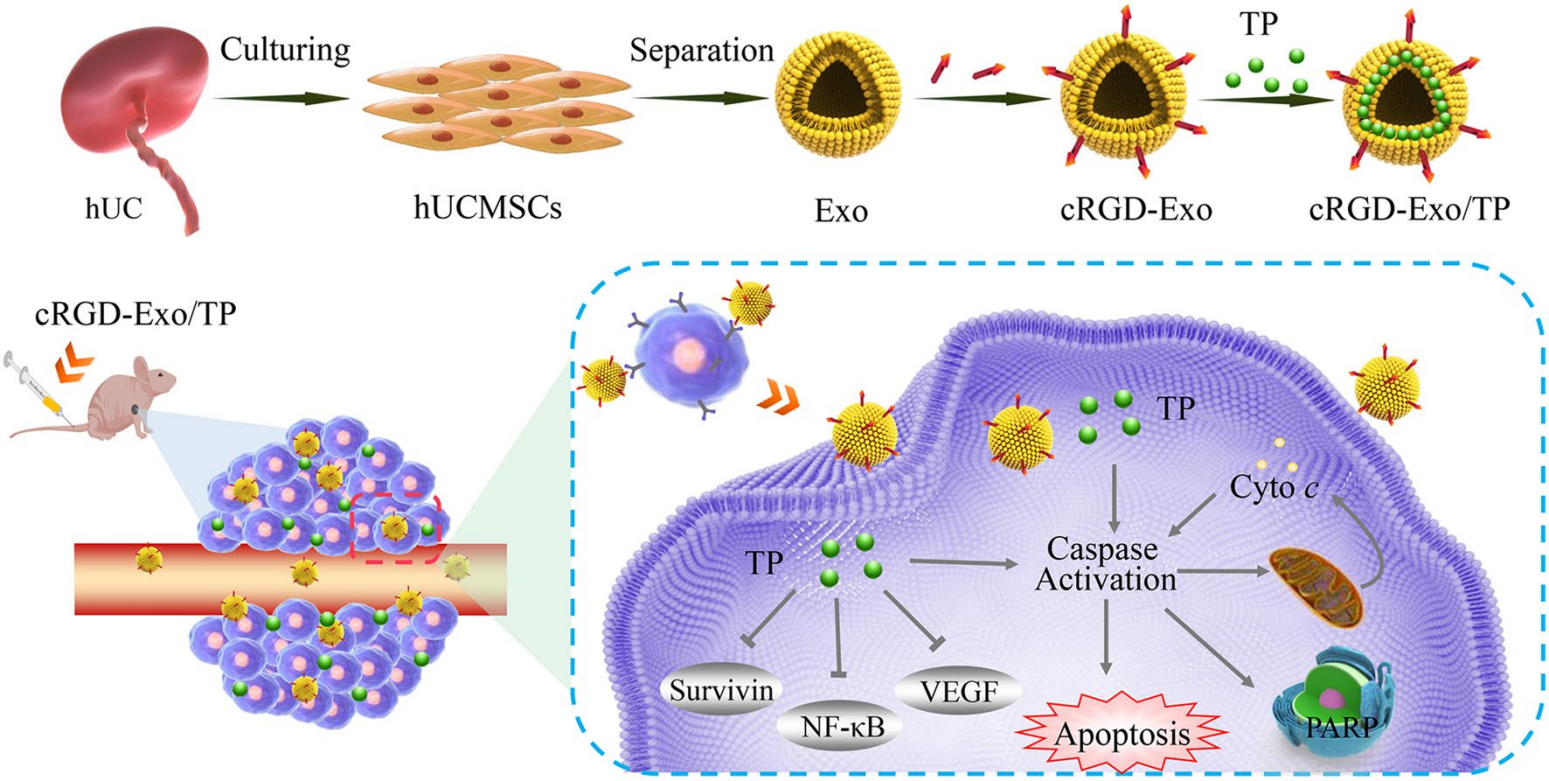

**Supplementary Information:**

The online version contains supplementary material available at 10.1186/s12951-022-01597-1.

## Background

Malignant melanoma (MM) is the most aggressive and fastest-growing skin tumor, accounting for 65% of skin cancer mortality [1]. The 5-year survival rate of patients with metastatic melanoma is less than 5% [2]. Chinese guidelines for diagnosis and treatment of melanoma [3] and the American Society of Clinical Oncology pointed out that only early MM can be surgically resected and that radiotherapy might be performed for inoperable primary or metastatic melanomas. However, radiotherapy has a negligible effect on survival time. In addition, the total effective rate of dacarbazine, the chemotherapeutic drug for MM approved by the FDA, is only 10–15% [3]. Although targeting drugs and monoclonal antibodies have certain efficacy, drawbacks such as high price, insufferable side effects, and drug resistance limit their further clinical application. Therefore, endeavors to scout new effective drugs and target delivery them to MM are urgently needed.

Recently, natural products have been proven to be appealing candidates for antitumor agents [4–6]. Among them, triptolide (TP), an epoxy diterpene lactone compound extracted from *Tripterygium wilfordii* Hook F, has been widely used in rheumatism, inflammation, immunosuppression, and anticancer treatment [7]. It has been confirmed to inhibit multiple tumors, including melanoma [8], pancreatic cancer [9], and breast cancer [10], by regulating cell proliferation, apoptosis, autophagy, and angiogenesis [11]. However, the clinical potential of TP is plagued by its aqueous insolubility, short half-life, and biotoxicity. Therefore, it is necessary to develop an ideal carrier for the targeted delivery of TP to tumor tissues to enhance efficacy and reduce toxicity.

Nanotechnology-based delivery systems are endowed with enhanced efficacy, alleviated adverse toxicity effects, and high entrapment capacity for drugs [12]. In recent decades, nanotargeted delivery systems have been shown to considerably reduce toxicity and prolong the circulation time of TP, achieving targeted delivery of therapies [13–15]. Meanwhile, carriers loading TP are still challenging: uncontrolled drug release before reaching lesions [16], rapid elimination by the mononuclear phagocytic system (MPS) [17], and unpredictable biosafety [18]. Fortunately, biomaterials offer alternative strategies as novel vehicles to overcome these drawbacks.

Exosomes (Exo) have received increasing attention as drug delivery systems due to their distinct properties. Exo, averaging 100 nm in diameter, can be endogenously secreted by multiple, if not all, types of cells and are widely present in body fluids [19, 20]. It plays “cellular postmen” roles in extra- and intercellular communication via the immanent cargos, such as proteins, lipids, and RNA [21, 22]. As naturally derived nanocarriers, Exo are characterized by low biotoxicity and immunogenicity and are especially structured with a lipid bilayer membrane and cargo protection ability [23–25]. However, most of the Exo naturally accumulated in the liver, spleen, and other normal organs through MPS after systemic administration [26]. Accordingly, it is necessary to artificially equip Exo with specific molecules for better tumor targeting [27, 28].

αvβ3 integrin overexpression on melanoma cells could mediate tumor angiogenesis through vascular endothelial growth factor (VEGF) and angiopoietin-Tie signaling pathways, suggesting that αvβ3 integrin is a promising therapeutic target [29, 30]. As reported, targeted therapeutic drugs have been developed based on antibodies, small molecules, and peptides [31]. For the last category, the recognition consequences of the peptide have been determined, and their preparation technology is more maneuverable. Among these, the cyclic arginine-glycine-aspartate peptide (cRGD) as a targeting ligand exhibits high affinity for the αvβ3 integrin receptor [32]. Encouragingly, this study attempted to employ cRGD to engineer Exo, aiming to construct a melanoma-targeting Exo nanoplatform.

As donor cells, mesenchymal stromal cells (MSCs) can not only be derived from almost all human tissues but also be highly proliferative to produce large-scale Exo [33, 34]. In particular, human umbilical cord MSCs (hUCMSCs) have attracted much attention due to their ease of access with few ethical considerations, simple culture, and rapid amplification [35].

In this study, Exo derived from hUCMSCs was engineered with cRGD, which targeted the αvβ3 integrin receptor overexpressed on the tumor cells, to develop the cRGD-Exo targeted delivery system. TP loaded in cRGD-Exo, namely, cRGD-Exo/TP, was prepared by coincubation with TP and cRGD-Exo. Subsequently, the mechanisms of cellular internalization, targeting capacity, and pharmacokinetics were explored. In addition, the antimelanoma potency of cRGD-Exo/TP was verified both in vitro and in vivo. Meanwhile, biosecurity was also evaluated after administration. The results implied that the developed Exo-based drug delivery system will provide a promising approach for the targeted delivery of TP to melanoma.

## Results and discussion

### Characterization of hUCMSCs

**Fig. 1:**
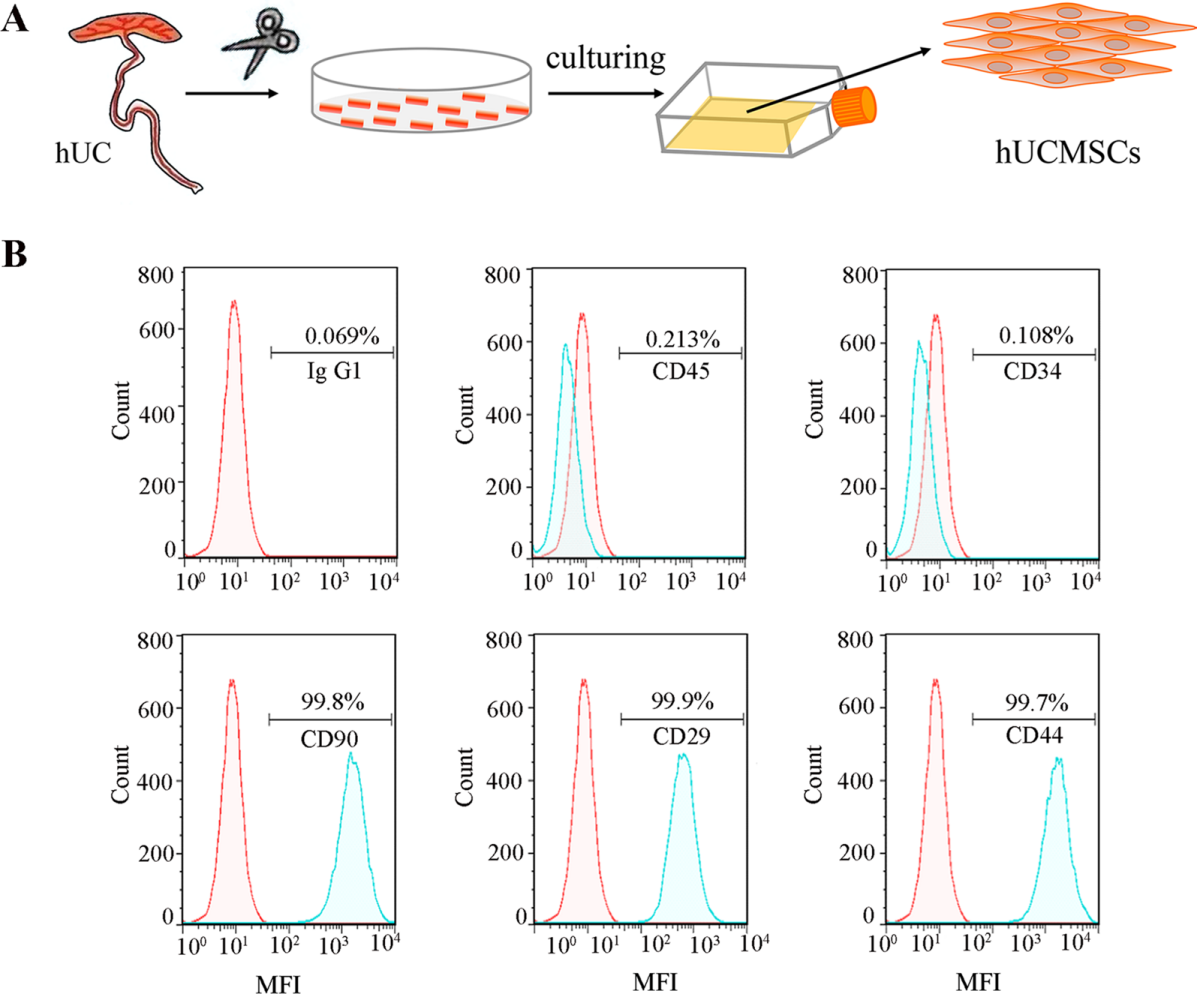
Extraction and characterization of hUCMSCs. **A** Schematic plot of the extraction of hUCMSCs from hUC. **B** Expression of hUCMSCs surface markers (CD45, CD34, CD90, CD29, CD44) identified by flow cytometry

A schematic of the extraction of hUCMSCs from hUC is illustrated in Fig. 1A. The cultured hUCMSCs were homogeneous and had long fusiform whirlpool arrangements under an inverted microscope (Additional file 1: Fig. S1). In addition, hUCMSCs surface markers were analyzed by flow cytometry (Fig. 1B). The positive rate of the MSC-negative markers CD45 and CD34 (expression in endothelial cells and haematopoietic stem cells) was less than 1%, while the positive rate of the markers CD90, CD29, and CD44 was more than 99%. These features of the cultured cells confirmed the representative characteristics of hUCMSCs [36]. The results indicated that hUCMSCs were successfully extracted from hUC and exhibited highly proliferative and self-renewal capabilities.

### Characterization of Exo, cRGD-Exo, and cRGD-Exo/TP

**Fig. 2:**
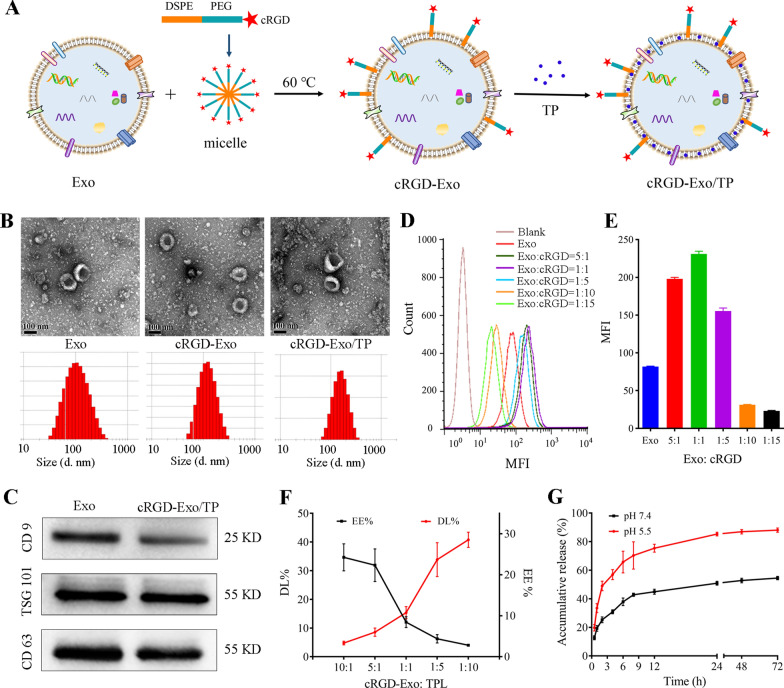
Preparation and characterization of Exo, cRGD-Exo, and cRGD-Exo/TP. **A** Schematic diagram of the construction of cRGD-Exo/TP. **B** The morphology and size distribution of Exo, cRGD-Exo, and cRGD-Exo/TP detected with TEM and dynamic light scattering, respectively. Scale bar: 100 nm. **C** The expression of Exo-specific protein markers (CD9, TSG101, and CD63) on Exo and cRGD-Exo/TP by western blotting. **D**, **E** The flow cytometry results and cellular MFI of A375 cells incubated with cRGD-Exo (different mass ratios of cRGD to Exo). **F** DL and EE of cRGD-Exo/TP with different mass ratios of cRGD-Exo to TP. **G** The drug release profiles of cRGD-Exo/TP in different pH release mediums for 72 h

Exo isolated from hUCMSCs were purified by gradient centrifugation. Then, cRGD-Exo was prepared by coincubation of Exo and self-assembled micelles (DSPE-PEG2000-cRGD) at 60 ℃. Then, TP was encapsulated in cRGD-Exo to construct the targeted drug delivery system cRGD-Exo/TP. The schematic diagram of the construction of cRGD-Exo/TP is displayed in Fig. 2A. The size, polydispersity index (PDI), and zeta potential of Exo were 82.85 ± 2.14 nm, 0.17 ± 0.03, and −21.20 ± 0.56 mV, respectively. The vesicles exhibited a distinct double-layer membrane-shaped saucer morphology, as shown by transmission electron microscopy (TEM, Fig. 2B and Additional file 1: Fig. S2). The Exo and cRGD mass ratio of cRGD-Exo was optimized by evaluating the tumor targetability of PKH67-labeled cRGD-Exo using a flow cytometer. The mean fluorescence intensity (MFI) of cRGD-Exo increased and then decreased with the addition of cRGD, which might be attributed to excess cRGD competitively binding to αvβ3 receptors on the A375 cell membrane. When the mass ratio of Exo to cRGD was 1:1, the MFI (231 ± 3.61) was the highest (Fig. 2D, E), which was significantly higher than that of the Exo group (MFI = 81.80 ± 0.56). After being engineered with cRGD, the morphology exhibited no obvious change, while the size of 108.77 ± 3.29 nm (PDI = 0.26 ± 0.04) tended to be larger than that of Exo (82.85 ± 2.14 nm). The cRGD-Exo zeta potential of −14.43 ± 0.42 mV increased compared with Exo (Additional file 1: Fig. S2), which might correspond to the incorporation of Exo membrane and cRGD micelles.

In addition, the TP content loaded in cRGD-Exo (cRGD-Exo/TP) was also screened by drug loading (DL) and encapsulation efficiency (EE). As shown in Fig. 2F, when the mass ratio of cRGD-Exo to TP was 1:1, cRGD-Exo/TP had higher EE and DL values of 12.07 ± 1.54% and 10.76 ± 1.21%, respectively. In addition, cRGD-Exo/TP was characterized by a larger hydrodynamic size of 157.34 ± 6.21 nm (with PDI of 0.28 ± 0.04) and a nonsignificant change in the zeta potential of −14.43 ± 0.42 mV compared with cRGD-Exo (Additional file 1: Fig. S2). In addition, the Exo-specific markers CD9, CD63, and TSG101 in Exo and cRGD-Exo/TP were confirmed by western blotting, which indicated that Exo was successfully isolated from hUCMSCs and that the structure of Exo was not disturbed by cRGD insertion and drug loading (Fig. 2C).

### Drug release

To investigate the release behaviors of cRGD-Exo/TP under normal physiological conditions and in the tumor microenvironment, the release curves were profiled in media with different pH values [37–39]. As shown in the release profiles (Fig. 2G), a burst release in the first 2 h was followed by a sustained release until 72 h. The initial fast release might be attributed to TP adhering to the cRGD-Exo surface. In addition, cRGD-Exo/TP showed controlled release profiles in different release media. The accumulated release rates of TP from cRGD-Exo/TP at pH 7.4 and pH 5.5 were 53.73 ± 1.18% and 87.31 ± 0.86% for 72 h, respectively, suggesting that cRGD-Exo might limit TP release in the blood circulation and normal tissue while effectively triggering drug release in the acidic tumor microenvironment. This might be because the acidic microenvironment could change the structure of Exo phospholipids, promoting drug release from the carrier [40]. The release pattern of cRGD-Exo/TP makes it a potential targeted drug delivery system for cancer treatment.

### Stability

The stability of cRGD-Exo/TP under different conditions was studied by monitoring the dynamic changes in particle size. The sizes of cRGD-Exo/TP in PBS (7 d) and Exo-free serum (24 h) during the studies were 155.30 ± 0.73 nm and 155.74 ± 0.62 nm, respectively. The negligible particle size variation during storage implied that cRGD-Exo/TP has good storage and serum stability (Additional file 1: Figs. S3, S4).

### In vitro internalization of cRGD-Exo/TP

**Fig. 3:**
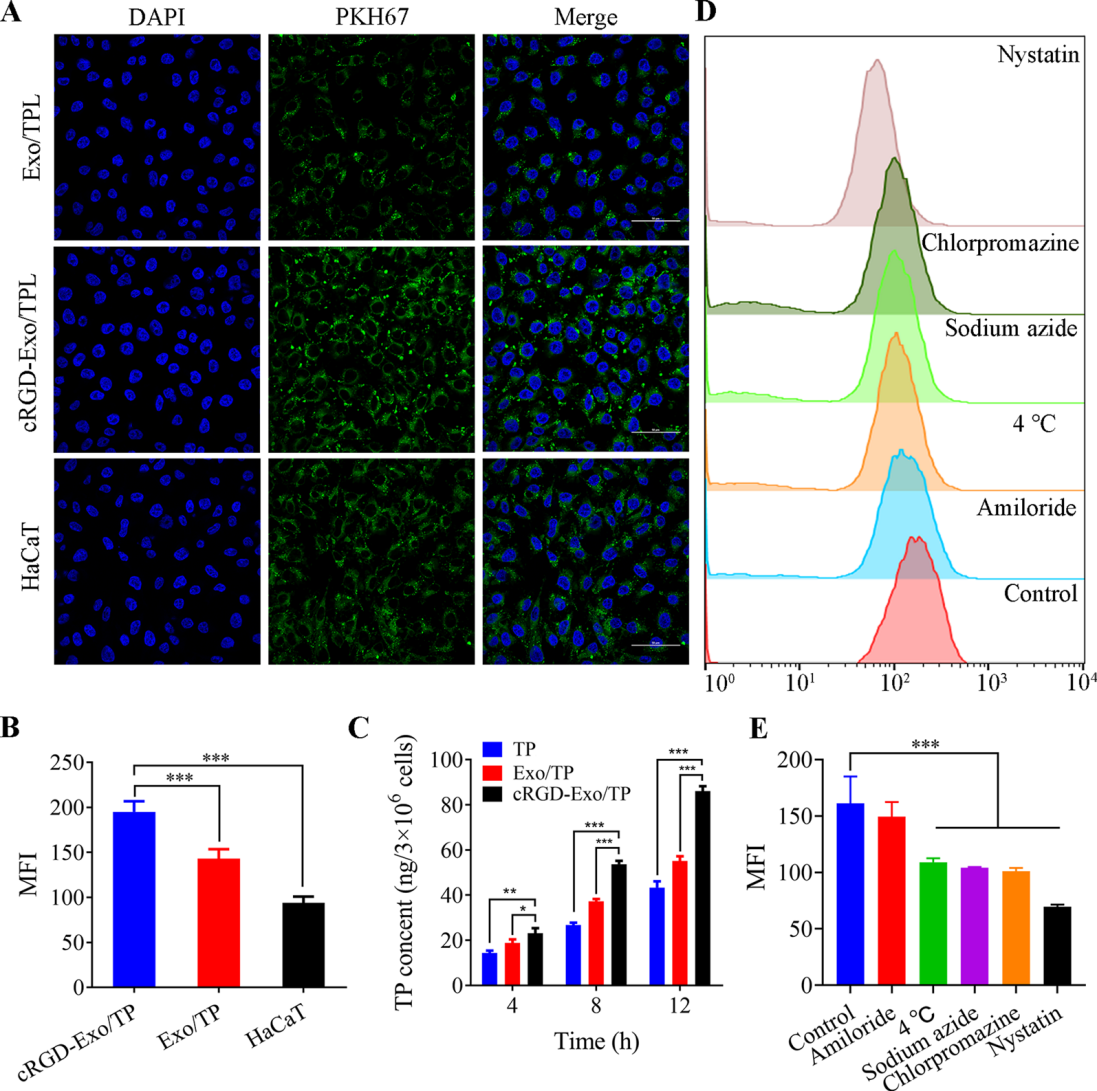
Cellular uptake and internalization mechanisms of cRGD-Exo/TP. **A** CLSM images of A375 cells incubated with Exo/TP, cRGD-Exo/TP, and HaCaT cells incubated with cRGD-Exo/TP for 4 h. Scale bar = 50 μm. **B** The MFI of A375 and HaCaT cells treated with PKH67-labeled Exo/TP or cRGD-Exo/TP. **C** Intracellular TP content was measured by UHPLC-MS/MS after the A375 cells were treated with free TP, Exo/TP, or cRGD-Exo/TP at different times. **D** Flow cytometry images of A375 cells treated with low temperature or different inhibitors. **E** The MFI of A375 cells pretreated with low temperature or different inhibitors. *p < 0.05, **p < 0.01, ***p < 0.001

Targetability is an important prerequisite for a targeted drug delivery system. As displayed in confocal laser scanning microscope (CLSM) images (Fig. 3A), cell nuclei (blue) and PHK67-labeled Exo (green) are observed. Colocalization images of PKH67-labeled Exo/TP or PKH67-labeled cRGD-Exo/TP and cells suggested that Exo/TP or cRGD-Exo/TP could be taken up into the cell cytoplasm to varying degrees. The semiquantitative results (Fig. 3B) coincided with CLSM, showing that the MFI in the cRGD-Exo/TP group was significantly higher than that in the Exo/TP group. The results indicated that internalization by A375 cells was mediated by αvβ3 integrin. In addition, the MFI of A375 cells was also significantly higher than that of human epidermal cells (HaCaT cells) (p < 0.01), which further confirmed the receptor-mediated cell-type specificity internalization of cRGD-Exo/TP (αvβ3 integrin protein was expressed at low expression on HaCaT cells compared with A375 cells, Additional file 1: Fig. S6) [41]. In addition, as shown in Fig. 3C, the cellular TP content in the cRGD-Exo/TP group was 1.98 times and 1.56 times higher than that in the Exo/TP and TP solution groups at 12 h, which might be due to Exo possessing excellent biocompatibility and cRGD guiding the specific targeting of cRGD-Exo/TP to αvβ3 integrin in melanoma cells [29, 42].

### Internalization mechanisms of cRGD-Exo/TP

Preconditioning cells with various inhibitors is an important means to study internalization mechanisms [43]. As shown in Fig. 3D, E, after pretreatment with sodium azide or low temperature, the MFI of A375 cells was reduced by 35.3% and 32.4%, respectively, suggesting that cRGD-Exo/TP was taken up by an energy-dependent active process. Upon pretreatment of A375 cells with chlorpromazine, the cellular MFI was reduced by 37.3% (p < 0.01). In addition, pretreatment of A375 cells with nystatin caused a more than 50% decrease in cellular uptake (p < 0.01). The results indicated that internalization was associated with clathrin-mediated and caveolin-dependent endocytosis. However, there was no significant difference in cellular uptake between the amiloride and control groups (p > 0.05), implying that macropinocytosis-mediated endocytosis is not involved in A375 cell uptake of cRGD-Exo/TP, which is consistent with previous studies [44, 45]. Upon reducing the incubation temperature or pretreatment with inhibitors, the internalization of cRGD-Exo/TP was markedly decreased to a certain extent compared with the control (at 37 °C), strongly indicating that multiple internalization pathways are involved.

### Cell proliferation inhibition

**Fig. 4:**
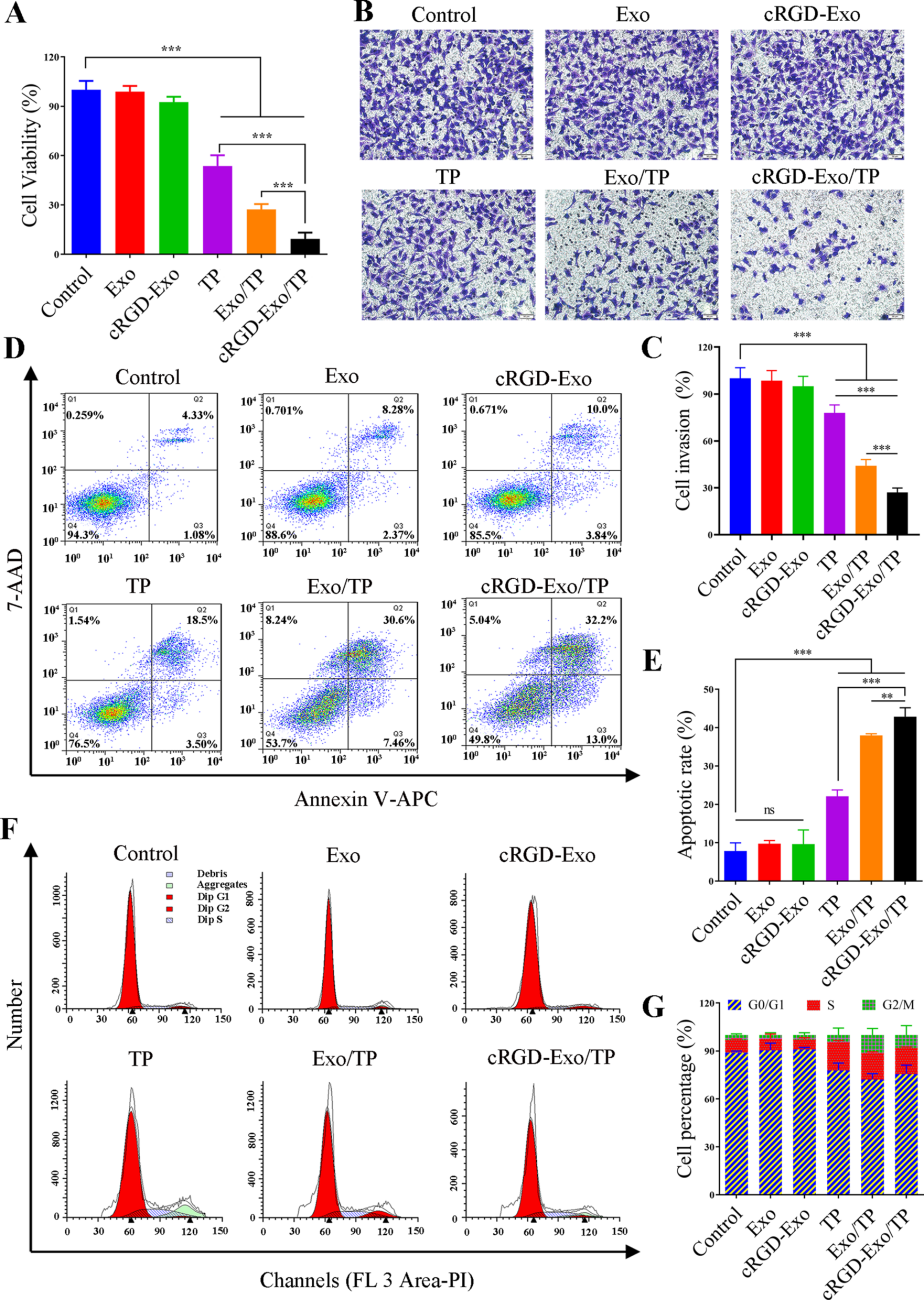
The in vitro antimelanoma efficacy. **A** Cell viability of A375 cells treated with Exo, cRGD-Exo, TP solution, Exo/TP, and cRGD-Exo/TP. The concentrations of TP and Exo (protein) were both 70 ng/mL. **B** Anti-invasion effects on A375 cells of different groups via Transwell assay. (Scale bar = 50 μm). **C** Quantitation of cell invasion results. **D** Flow cytometry analysis of apoptosis. **E** Quantitative analysis of the cell apoptotic rate using ImageJ software. **F** The cell cycle distribution of different groups. **G** Quantitative analysis of the cell cycle distribution. *p < 0.05, **p < 0.01, ***p < 0.001

The antiproliferative ability of cRGD-Exo/TP against A375 cells was investigated using a CCK-8 assay. TP showed concentration-dependent cytotoxicity, and the IC50 was 69.63 ng/mL (Additional file 1: Fig. S5). In addition, Fig. 4A shows the viability of the cells treated with DMEM, Exo, cRGD-Exo, TP solution, Exo/TP, and cRGD-Exo/TP. Exo and engineered cRGD-Exo showed negligible cytotoxicity, while free TP, Exo/TP, and cRGD-Exo/TP all inhibited cell proliferation. In addition, the cytotoxicity of TP was enhanced after being encapsulated in Exo and further enhanced after being encapsulated in cRGD-Exo. This might be because Exo derived from cells with high biocompatibility, as natural nanocarriers, have the ability to penetrate biological barriers carrying the loading drugs. On the one hand, Exo could deliver TP into tumor tissue via the EPR effect, and cRGD-Exo/TP was endowed with higher tumor-targeting ability after being engineered with cRGD, further enhancing the interaction with tumor cells. On the other hand, TP might be effluxed by the P-glycoprotein (P-gp) of A375 cells [46, 47]. The results were consistent with those of cell uptake.

### Invasion suppression

Tumor cell invasion is closely related to cancer progression. Therefore, we also studied the anti-invasion effect of cRGD-Exo/TP via a Transwell assay. Serum (10%) added to the bottom chamber for stimulated haptotaxis motility was introduced to evaluate the anti-invasion ability of cRGD-Exo/TP on A375 cells [45]. Microscopic photographs (Fig. 4B) and migrated cell amounts (Fig. 4C) indicated that TP solution, Exo/TP, and cRGD-Exo/TP could significantly inhibit A375 cell invasion with invasion inhibition rates of 45.54 ± 6.35%, 62.48 ± 4.38%, and 78.93 ± 4.13%, respectively. The results might be attributed to cRGD-Exo/TP decreasing A375 cell penetrability and chemotactic ability [48].

### Cell apoptosis promotion

The flow cytometry results of the apoptosis assay (Fig. 4D, E) confirmed that Exo caused minimal apoptosis and necrosis in A375 cells (11.03 ± 0.86%), which was similar to that of the cRGD-Exo group (12.63 ± 1.05%). TP/Exo solution induced cellular apoptosis (30.6%) and necrosis (7.46%). The mortal cells were higher than those of the TP groups (22.10 ± 1.66%). As expected, 32.2% of apoptotic cells and 13.0% of necrotic cells were detected in the cRGD-Exo/TP group.

### Cell cycle arrest

PI staining was carried out to investigate whether TP could induce cell cycle alterations. As shown in Fig. 4F, G, when treated with TP solution, Exo/TP or cRGD-Exo/TP, the cell population of the G_0_/G_1_ phase decreased from 88.91 ± 0.81% (control) to 72.87 ± 3.71%, 72.17 ± 3.00%, and 75.58 ± 4.57%, respectively, while the S phase increased from 8.07 ± 0.39% (control) to 17.73 ± 0.79%, 16.61 ± 0.53%, and 16.35 ± 0.42%, respectively. There was no significant difference between TP solution and TP-encapsulated Exo. These results indicated that TP mainly arrested the S phase of the cell cycle and thereby affected mitosis and inhibited tumor cell growth.

### Mechanisms of apoptosis

**Fig. 5:**
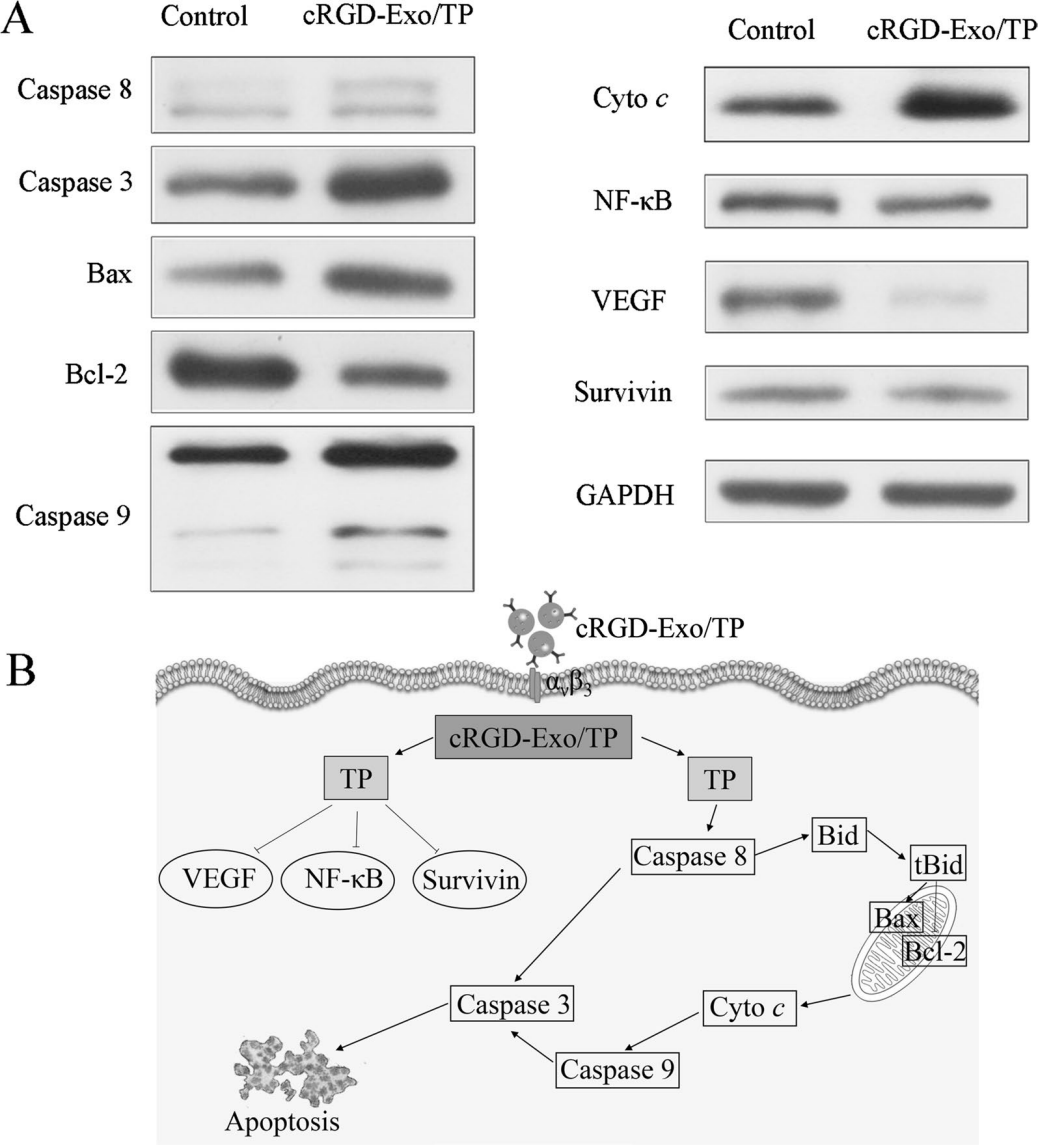
The mechanisms of cRGD-Exo/TP inducing tumor cell apoptosis. **A** Western blotting analysis of apoptosis-related protein expression in A375 cells. **B** Schematic diagram of the TP-induced apoptosis pathway. The arrowhead indicates the promotion of related protein expression, and the blunt arrowhead indicates the inhibition

Based on the results that cRGD-Exo/TP effectively inhibited cell proliferation and invasion and promoted cell apoptosis, we further explored the molecular mechanisms at the cellular level. As reported, cell apoptosis is related to the death receptor-dependent pathway (extrinsic pathway) and mitochondrial-dependent pathway (intrinsic pathway) [49]. Figure 5A shows that cRGD-Exo/TP significantly increased the expression of caspase-8 and activated caspase-3, which indicated that the extrinsic pathway was involved in apoptosis. In addition, Bcl-2 family proteins include proapoptotic proteins such as Bax and antiapoptotic proteins such as Bcl-2. The increased ratio of Bax/Bcl-2 could consecutively trigger the expression of cyto *c* and caspase-9, leading to mitochondrial dysfunction through the caspase cascade and mitochondrial pathways [50]. Additionally, Survivin, VEGF, and NF-κB are closely related to the occurrence, angiogenesis, proliferation, metastasis, apoptosis, and drug resistance of melanoma [51–53]. The reduced expression of survivin, VEGF, and NF-κB implied that cRGD-Exo/TP inhibited the growth of A375 cells by inhibiting tumor angiogenesis, cell proliferation, and migration. The potential apoptosis pathways are shown in Fig. 5B.

### In vivo biodistribution and pharmacokinetics of cRGD-Exo/TP

**Fig. 6:**
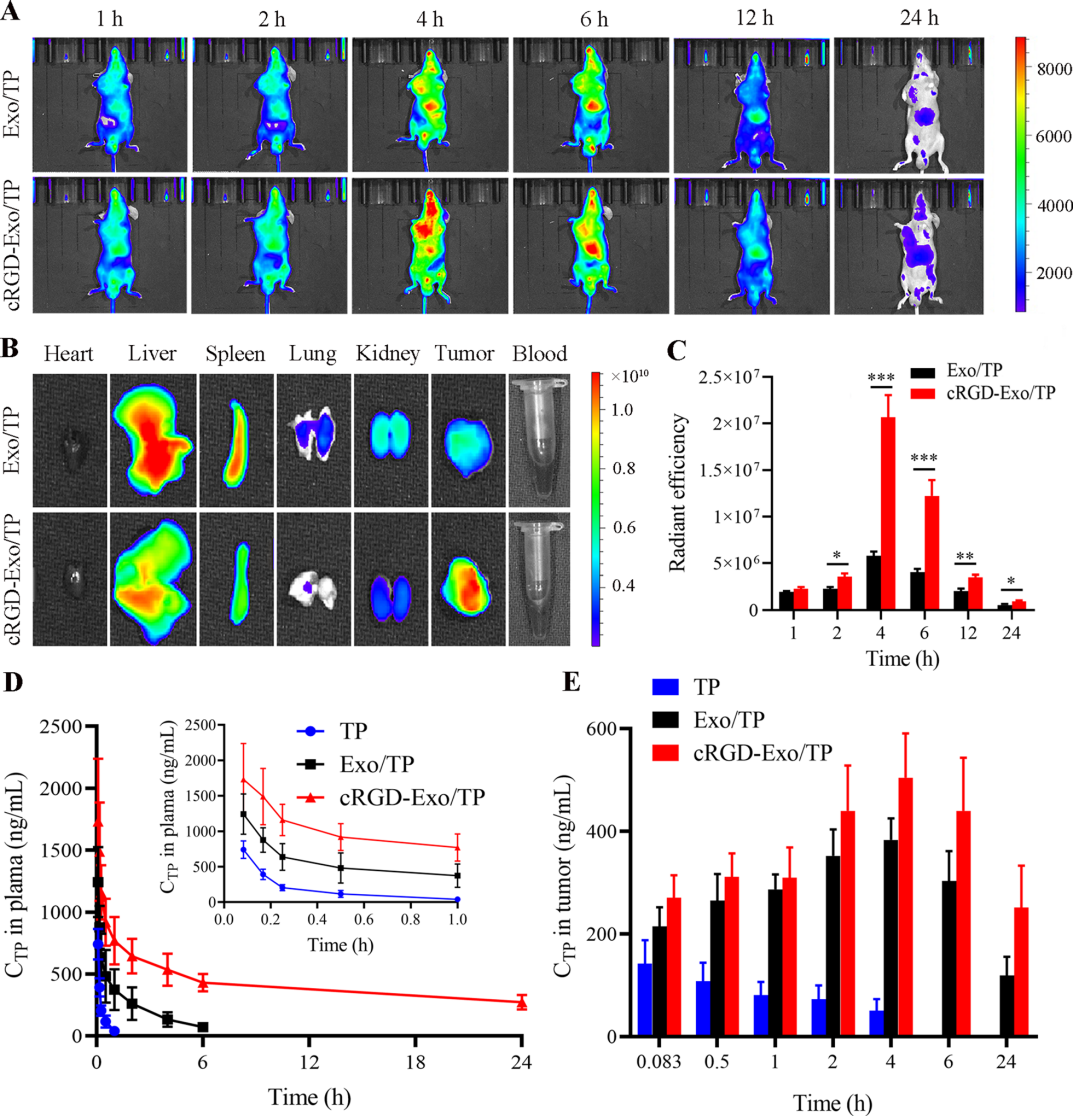
In vivo biodistribution and pharmacokinetics of cRGD-Exo/TP. **A** The in vivo fluorescence images of tumor-bearing mice treated with DiR-labeled Exo/TP or DiR-labeled cRGD-Exo/TP for 24 h. **B** The *ex-vivo* fluorescence images of primary organs, tumor tissues, and blood captured at 24 h after administration. **C** The fluorescence intensity in tumor tissues at different times. **D** TP concentration–time profiles of the TP solution, Exo/TP, and cRGD-Exo/TP groups in tumor-bearing mice. **E** TP concentration in tumor tissues at predetermined times of TP solution, Exo/TP, and cRGD-Exo/TP groups. *p < 0.05, **p < 0.01, ***p < 0.001

The longitudinally dynamic distribution of Exo/TP and cRGD-Exo/TP labeled with DiR was tracked by an in vivo imaging system (IVIS) based on a timeline. As displayed in Fig. 6A, the fluorescence intensity in mice of the two groups was steadily enhanced within 4 h, and then the signal gradually faded. In addition, DiR-cRGD-Exo/TP could better target and accumulate in the tumor site, exhibiting that the fluorescence signal peak value of the cRGD-Exo/TP group was higher than that of the Exo/TP group. Furthermore, a stronger fluorescent signal in the tumoral and peritumoral areas was visualized in the cRGD-Exo/TP group than in the Exo/TP group within 24 h after injection. The ex vivo fluorescence intensity of excised primary organs, blood, and solid tumors was also detected after 24 h of administration to evaluate the tumor-targeting capacities of cRGD-Exo/TP. As shown in Fig. 6B, similarly, the fluorescence signal in the tumor tissue of cRGD-Exo/TP-treated mice was stronger than that of the Exo/TP group. In addition, fluorescence signals can also be observed in the liver, spleen, and kidney, whereas a relatively weak signal was detected in the lungs and heart and a negligible signal was detected in blood. For semiquantitative analysis, Fig. 6C illustrates the fluorescence intensities in tumor tissue of the two groups over time, which intuitively showed the longer retention time and better tumor targeting of cRGD-Exo/TP.

Quantitative analysis of TP concentration in blood, primary organs, and tumor tissues was carried out to investigate the pharmacokinetics and biodistribution of TP solution, Exo/TP, and cRGD-Exo/TP. The mean plasma concentration–time curves of tumor-bearing mice are shown in Fig. 6D. The main pharmacokinetic parameters fitted by the noncompartment model are shown in Table 1. As shown, the t_1/2_ of cRGD-Exo/TP in plasma was 27.14 ± 2.55 h, which was 11.61 times and 87.65 times higher than that of Exo/TP and TP solution, respectively. The prolonged half-time of TP after it was encapsulated in Exo or cRGD-Exo might be due to the Exo membrane preventing the encapsulated drug from untimely leakage, delaying elimination in the bloodstream, and prolonging the circulation time in vivo [48].

**Table 1:** Pharmacokinetic parameters of TP solution, Exo/TP, and cRGD-Exo/TP

Parameters	Unit	cRGD-Exo/TP	Exo/TP	TP (solution)
AUC_∞_	ng/mL*h	21,137.30 ± 4578.44	1767.83 ± 552.66	1400.43 ± 81.44
Cmax	ng/mL	1731.79 ± 413.55	1241.93 ± 232.39	612.44 ± 100.02
t_1/2_	h	27.14 ± 2.55	2.337 ± 0.34	0.31 ± 0.06
MRT	h	36.57 ± 3.35	2.80 ± 0.29	0.06 ± 0.03
CL	mL/h/kg	151.45 ± 30.86	1904.31 ± 564.64	2193.14 ± 124.42

In addition, the in vivo biodistribution trends of cRGD-Exo/TP and Exo/TP were consistent with that of the fluorescence signal by IVIS. For targeted analysis, the TP concentration in tumor tissue was detected over time. Compared with the cRGD-Exo/TP and Exo/TP groups, the lower accumulation of free TP in the tumor might be attributed to the rapid clearance and short half-time in blood [54], and Exo could certainly accumulate and penetrate the tumor site as a nanobiological carrier [55, 56]. In addition, the intertumoral TP accumulation of cRGD-Exo/TP was 1.32 times higher at 4 h and 2.11 times higher at 24 h than that of Exo/TP (Fig. 6E), which might be due to cRGD-Exo/TP showing outstanding targetability to the melanoma mouse model and reducing nonspecific capture from the liver, spleen, and other organs relying on cRGD engineering [57, 58]. Conversely, the nonspecific accumulation of cRGD-Exo/TP in primary organs was significantly lower than that of Exo/TP and TP solution (Additional file 1: Fig. S7). Compared with normal tissues, an acidic tumor microenvironment spontaneously facilitated cellular uptake and intratumoral penetration, which also accounted for the phenomenon that cRGD-Exo/TP was highly distributed in tumor sites and was low in normal tissues [59, 60].

### In vivo antitumor efficacy

**Fig. 7:**
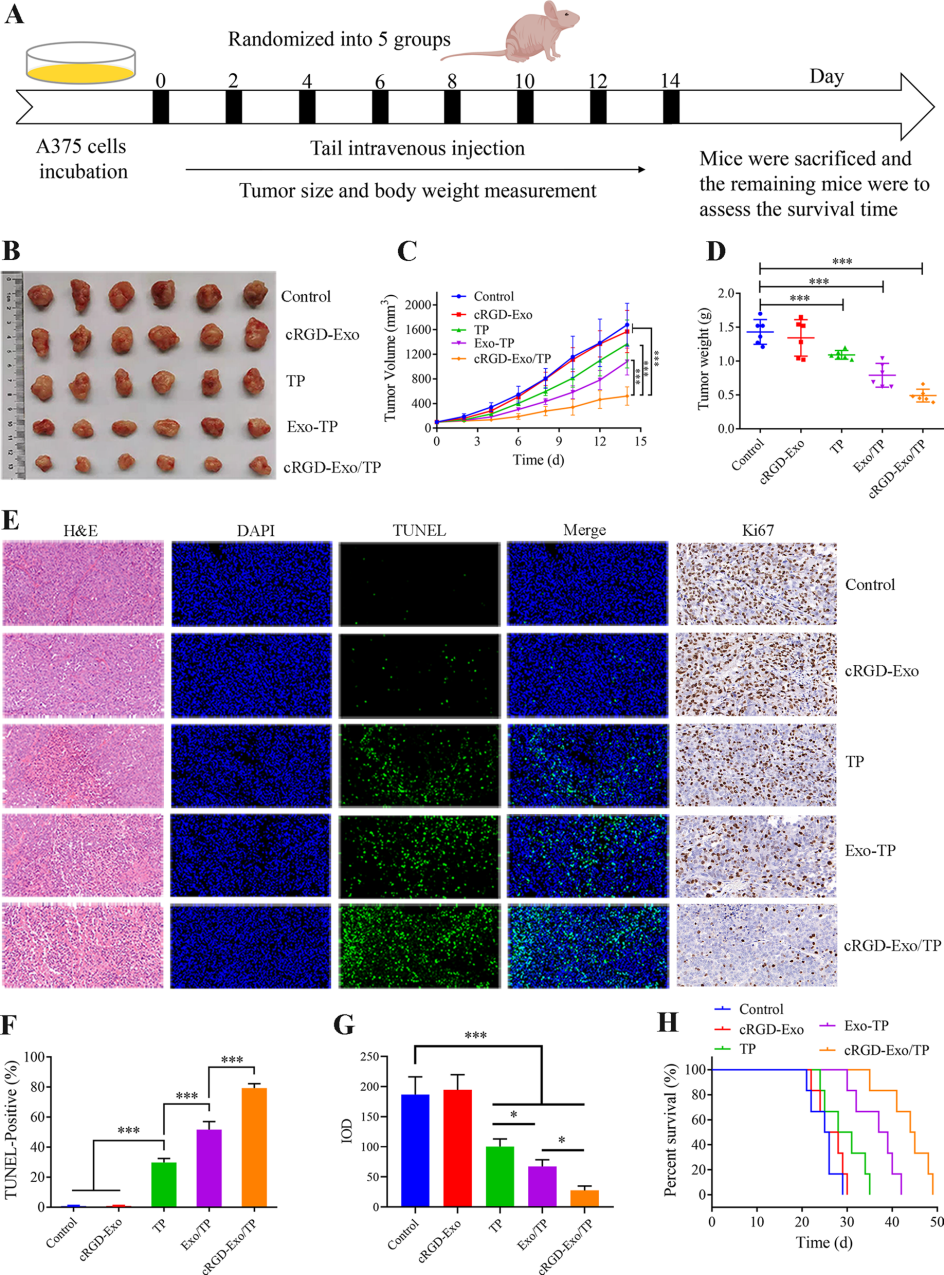
Anti-melanoma efficacy in vivo. **A** Schematic diagram of animal grouping and manipulations. **B** Representative images of the harvested xenograft tumors of the normal saline, cRGD-Exo, TP solution, Exo/TP, and cRGD-Exo/TP groups. **C** Tumor volume of different groups at interval times. **D** Tumor weight of different groups at the endpoint of the experiment. **E** H&E (scale bar: 50 μm), TUNEL (scale bar: 50 μm), and Ki67 (scale bar: 20 μm) results of tumor tissues in different groups. **F** Semiquantitative analysis of TUNEL-positive cells. **G** Semiquantitative analysis of IOD in Ki67 staining; **H** The survival time of tumor-bearing mice with different treatments. *p < 0.05, **p < 0.01, ***p < 0.001

Encouragingly, cRGD-Exo/TP exhibits excellent in vitro antitumor effects and in vivo tumor targeting. Therefore, we further investigated the in vivo antimelanoma efficacy of cRGD-Exo/TP in tumor-bearing mice. The procedure of the experiments is diagramed in Fig. 7A. As shown in Fig. 7B, C, the tumor growth of the cRGD-Exo/TP group was significantly suppressed compared with that of the other groups. Similarly, the tumor weight tendency at the endpoint of the experiment was consistent with the tumor volume variations (Fig. 7D), with tumor inhibition rates of 5.08 ± 20.39%, 6.18 ± 20.39%, 44.76 ± 9.33%, and 65.73 ± 3.29% in the cRGD-Exo, TP solution, Exo/TP, and cRGD-Exo/TP groups, respectively.

As histopathological examination (H&E, hematoxylin–eosin) staining shows in Fig. 7E, the pathological features of tumor sections, such as shrinking nuclei, focal necrosis, and more cytoplasm, could be visualized in the cRGD-Exo/TP groups. Terminal-deoxynucleotidyl transferase-mediated nick end labeling (TUNEL) further demonstrated that positive cells (apoptotic cells, green fluorescence) were notably increased after treatment with cRGD-Exo/TP compared with the control, cRGD-Exo, TP solution, and Exo/TP groups (Fig. 7E, F). In addition, the results of Ki67 staining (Fig. 7E, G) also indicated that cRGD-Exo/TP could significantly inhibit tumor cell proliferation with the lowest Ki67-positive staining (integrated optical density (IOD) = 27.66 ± 5.85%).

The survival time of tumor-bearing mice was profiled to confirm the long-term efficacy of cRGD-Exo/TP (Fig. 7H). The Kaplan–Meier survival curves indicated that the mean survival time of the mice treated with cRGD-Exo/TP was significantly extended compared with that of the other groups (44.5 days in the cRGD-Exo/TP group *vs* 25.5 days in the control group *vs* 26.5 days in the cRGD-Exo group vs 29.5 days in the TP group *vs* 38 days in the Exo/TP group).

The results indicated that cRGD-Exo/TP had an overwhelming inhibitory effect on melanoma. On the one hand, Exo naturally carrying body fluids such as blood and tissue fluid are moving continuously and can interact specifically with targeted cells in vivo, especially, Exo engineered with cRGD; on the other hand, the acidic tumor microenvironment could change the Exo intratumoral permeability and Exo membrane stabilization, which might drive cRGD-Exo/TP to elicit prominent efficacy [61, 62].

### In vivo biosafety evaluation of cRGD-Exo/TP

**Fig. 8:**
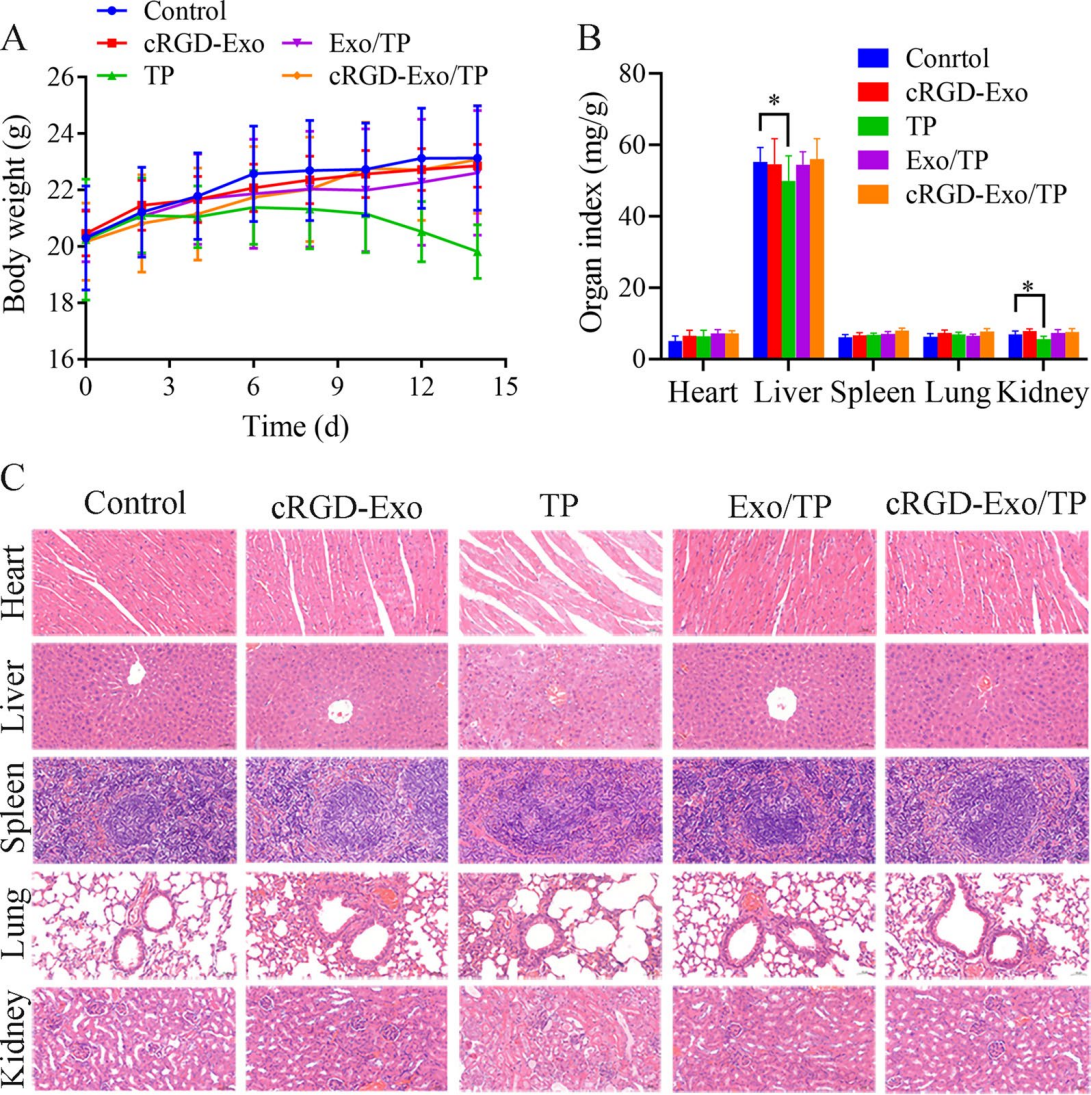
Biosafety evaluation of cRGD-Exo/TP. **A** The body weight of tumor-bearing mice treated with normal saline, cRGD-Exo, TP solution, Exo/TP, and cRGD-Exo/TP. **B** The major organ index of tumor-bearing mice with different treatments. **C** H&E staining (200×) of primary organs harvested from the different treated mice. *p < 0.05

Considering that TP is effective in the treatment of cancer accompanied by serious side effects, we evaluated the biosafety of cRGD-Exo/TP by monitoring the body weight changes of mice during the treatment period, organ index, H&E staining of normal tissues, routine blood tests, hepatic function tests, and renal function tests. As shown in Fig. 8A, the mice bodyweight of the cRGD-Exo/TP and Exo/TP groups increased slowly and was not significantly different from that of the control and cRGD-Exo groups. However, the mice of the TP solution group suffered a bodyweight loss. In addition, as shown by the organ indexes (Fig. 8B), the liver and kidney indexes of the free TP-treated mice were significantly lower than those of the control mice (p < 0.05). Fortunately, no obvious changes in organ indexes were observed between the cRGD-Exo/TP and control groups. Meanwhile, H&E staining images showed that the pathological differences between the cRGD-Exo/TP group and the control group were negligible. In addition, the heart, liver, and kidney were damaged in the TP solution group (Fig. 8C): cardiac muscle cells dilated and became congested; the liver tissues showed serious cell disorder, vacuolar degeneration, and focal necrosis; and the endothelial cells of the glomerulus and renal tubes were significantly abnormally swollen. In addition, as shown in Table 2, there was no significant difference in the parameters of red blood cells (RBC) and white blood cells (WBC) of the cRGD-Exo/TP group compared with the control group, while the WBC index was upregulated in the TP solution group. For the hepatic and renal function tests, the values of alanine aminotransferase (ALT) and creatinine (Cr) in the cRGD-Exo/TP group were not significantly different, whereas they were increased in the TP solution group. The biosafety results reconfirmed the in vivo biocompatibility of cRGD-Exo/TP, which might contribute to the tumor targeting and low distribution in normal tissues.

**Table 2:** The hematological parameters of the tumor-bearing mice with different treatments. (*n* = 5)

Parameters	RBC^a^ (× 10^12^/L)	WBC^b^ (× 10^9^/L)	ALT^c^ [U/L]	Cr^d^ [μmol/L]
Groups				
Control	10.00 ± 0.76	5.12 ± 0.56	65.97 ± 5.03	20.90 ± 1.98
cRGD-Exo	10.52 ± 0.83	5.46 ± 0.47	66.43 ± 5.83	20.77 ± 2.05
TP solution	8.11 ± 0.65**	6.73 ± 0.51**	76.25 ± 4.03*	27.76 ± 1.52**
Exo/TP	10.31 ± 0.62	4.92 ± 0.38	66.92 ± 2.47	19.63 ± 0.80
cRGD-Exo/TP	10.07 ± 0.68	5.55 ± 0.31	63.19 ± 3.54	20.41 ± 1.69

^a^Red blood cell

^b^white blood cell

^c^alanine aminotransferase

^d^creatinine

*p < 0.05

**p < 0.01 versus the control

## Conclusion

In summary, Exo was successfully extracted from hUCMSCs derived from hUC and engineered with cRGD based on the αvβ3 integrin. cRGD-Exo loading TP, namely, cRGD-Exo/TP, could be taken up by A375 cells via multiple internalization pathways. In addition, cRGD-Exo/TP significantly inhibited proliferation, invasion, and apoptosis promotion in vitro by disturbing the caspase cascade and mitochondrial pathways and altering the cell cycle distribution. Furthermore, the in vivo biodistribution and pharmacokinetics indicated that cRGD-Exo/TP possessed an excellent melanoma-targeting ability and prolonged the half-life of TP. In addition, in vivo antitumor results demonstrated that cRGD-Exo/TP significantly inhibited tumor growth and extended the survival time of tumor-bearing mice with negligible biotoxicity. Collectively, the research proposed a prototype melanoma-targeting delivery system for hydrophobic drugs, which provides a prospective strategy for the antitumor field.

## Experimental section

### Materials and methods

#### Materials

The materials used in this study were as follows: 1,2-distearoyl-sn-glycerol-3-phosphoethanolamine-[(polyethylene glycol)-2000]-c(RGDfK) (DSPE-PEG2000-cRGD) synthesized by Xi’an ruixi Biological Technology Co., Ltd. (Xi’an, China); 4-(2-hydroxyethyl)-1-piperazineethanesulfonic acid buffer (HEPES); CD63 (Abcam, 1: 1000, UK); CD9 (Abways 1:1000, China); TSG101 (Abways 1:1000, China); Cell Counting Kit-8 (CCK-8, Yeasen Bio, China); Annexin V-APC/7-AAD Apoptosis Detection Kit (YEASEN, China); 4′,6-diamidino-2-phenylindole (DAPI, Beyotime, China); PKH67 (Sigma, USA); DiR (Biotium, USA); Dulbecco’s modified Eagle’s medium (DMEM); Fetal bovine serum (FBS), Trypsin and 1% Pen-Strep (Thermo Fisher Scientific, Waltham, USA). Unless otherwise stated, other reagents and solvents were purchased from commercial sources and can be used without further purification.

#### Cell culture

hUCMSCs were derived from the umbilical cord of healthy newborns delivered by cesarean section. The protocols were approved by the Institutional Review Board at Changhai Hospital of Naval Medical University. A375 and HaCaT cells were purchased from the Chinese Academy of Sciences Cell Bank (Shanghai, China) and cultured in DMEM containing 10% FBS and 1% Pen-Strep under a 5% CO_2_ atmosphere at 37 °C.

#### Animals

Male BALB/c nude mice (18–22 g) provided by Fudan University Shanghai Cancer Center were housed under standard laboratory conditions (25 °C, 50–60% humidity on 12 h light/dark cycles) with free access to water and standard chow.

#### Culturing and characterization of hUCMSCs

Culturing of hUMSCs was carried out based on previous methods [63]. Briefly, the hUC of healthy newborns delivered by cesarean section was collected, washed with DMEM and 70% ethanol, and then cut into small tissue masses (2–4 mm). Then, the tissue masses were evenly arranged in the culture dish with gaps left and incubated with a standard culture medium at 37 °C. When the fibroblast-like cells (hUCMSCs) climbed out from the edge of the tissue masses and reached 70–80% confluence, the cells were trypsinized and prepared for subculture. The typical protein markers (CD45, CD34, CD90, CD29, and CD44) of hUCMSCs at passage 3 were detected by a flow cytometer (BD Biosciences, USA) [64, 65].

#### Isolation and characterization of hUCMSCs-Exo

Exo was isolated from passages 3–10 hUCMSCs by gradient centrifugation [28]. When hUCMSCs reached 70%-80% confluence, the cells were washed with PBS and cultured with 10% Exo-depleted FBS for 12 h. The harvested supernatant was ultracentrifuged at 300× *g* for 10 min, 2000× *g* for 10 min, and 10,000× *g* for 30 min at 4℃ to remove cells and cell debris. The supernatants were then ultracentrifuged at 120,000× *g* for 70 min. The pellet was resuspended in PBS and ultracentrifuged again at 120,000× *g* for another 70 min. Finally, hUCMSC-Exo pellets were resuspended in PBS and stored at −80 °C for further use.

The morphology, size, size distribution, and zeta potential of hUCMSCs-Exo were detected using TEM (Hitachi, Tokyo, Japan) and dynamic light scattering (DLS, Zetasizer Nano ZS90, Malvern, UK). In addition, western blotting analysis was implemented to identify the exosomal markers CD9, CD63, and TSG101 of hUCMSCs-Exo. Briefly, the total protein of hUCMSCs-Exo was detected by a BCA kit, separated on a 10% sodium dodecyl sulfate–polyacrylamide gel, and then transferred onto polyvinylidene fluoride membranes. After blocking with 5% skimmed milk, the membranes were incubated with CD9, CD63, and TSG101 primary antibodies overnight and horseradish peroxidase-conjugated (HRP) secondary antibodies. Finally, the immunoblots on the membrane were detected by enhanced chemiluminescence reagents (Amersham Pharmacia Biotech, Little Chalfont, UK) [42, 66].

DSPE-PEG2000-cRGD was incorporated into Exo by the postinsertion method [42, 67]. Briefly, cRGD was dissolved in HEPES for 15 min at 60 °C to form micelles. The micelles were sonicated to reduce the size to facilitate their separation from Exo. Exo labeled with PKH67 were mixed with the formed micelles at mass ratios (μg protein:μg protein) of 5:1, 1:1, 1:5, 1:10, and 1:15 for 2 h at 40 °C. After that, A375 cells were treated with the above prepared PKH67-labeled cRGD-Exo for 6 h. Then, the treated cells were washed, trypsinized, and resuspended in PBS for flow cytometry analysis to screen the ratio of cRGD and Exo based on fluorescence intensity. After that, the optimized Exo and cRGD micelles were mixed at 40 °C for 2 h and then exposed to size-exclusion chromatography to purify cRGD-Exo.

#### Preparation and characterization of cRGD-Exo/TP

The coincubation method was applied to load TP into cRGD-Exo. First, purified cRGD-Exo (600 μg/mL) and TP (40 μg/mL) at different mass ratios (μg protein:μg TP) of 10:1, 5:1, 1:1, 1:5, and 1:10 were mixed and incubated in a shaker at 100 rpm for 1 h at room temperature to prepare drug-encapsulated exosomes, cRGD-Exo/TP. The unloaded drug was quantitated by HPLC to calculate DL and EE by the following Eqs. 1 and 2. Exo/TP was prepared with the optimized prescription of cRGD-Exo/TP. The morphology, size, size distribution. and zeta potential of Exo/TP and cRGD-Exo/TP were also characterized.

1 $${\text{DL }}\left( \% \right) \, = \, \left( {{\text{Wt}} - {\text{Wf}}} \right)/{\text{Wm}} \times {1}00\%$$

2 $${\text{EE }}\left( \% \right) \, = \left( {{\text{Wt}} - {\text{Wf}}} \right)/{\text{Wt}} \times {1}00\%$$

where Wt, Wf, and Wm represent the weight of total TP added, the weight of the unloaded TP, and the total protein weight of cRGD-Exo, respectively.

#### Drug release assay

The in vitro drug release behavior of cRGD-Exo/TP was analyzed using the dialysis method [68]. Briefly, 2 mL of cRGD-Exo/TP solution was transferred into a dialysis bag immersed in pH 7.4 or pH 5.5 PBS (containing 20% ethanol for sink conditions) maintained at 37 ± 0.5 °C under 100 rpm shaking. 1 mL of release medium was sampled and immediately replaced with fresh medium at 0.5, 1, 2, 4, 6, 8, 12, 24, 48, and 72 h for HPLC analysis to calculate the cumulative release percent. The cumulative drug release rate was calculated with the following Eq. (3):

3 $$E\% = \left[ {\frac{{c_{n} }}{{{L \mathord{\left/ {\vphantom {L {v_{2} }}} \right. \kern-\nulldelimiterspace} {v_{2} }}}} + \frac{{\left( {c_{n - 1} + \cdots c_{2} + c_{1} } \right)v_{1} }}{L}} \right] \times 100\%$$

where, the E% represents the accumulated release rate, C*n* represents the TP concentration of *the n*th sample, L represents the TP content of cRGD-Exo/TP added, and ν_1_ and ν_2_ represent the volumes of the sample and receiver medium, respectively.

#### Stability study

Storage stability and serum stability were assessed by monitoring the particle size of cRGD-Exo/TP in PBS (4 °C, 7 days) or in 10% Exo-free serum (37 °C, 24 h). The size of cRGD-Exo/TP in PBS was detected every day, and that in serum was detected at 2, 4, 6, 8, 10, 12, and 24 h.

#### In vitro internalization of cRGD-Exo/TP

A375 and HaCaT cells were exposed to PKH67-labeled Exo/TP or cRGD-Exo/TP for 4 h, after which the cells were fixed with 4% paraformaldehyde and stained with DAPI. When the samples were dried at room temperature in the dark, the cellular fluorescence intensity was observed with CLSM (Leica, Germany). In addition, for semiquantitative analysis, the intracellular MFI of the cells with different treatments was calculated with ImageJ software.

Furthermore, TP taken up by A375 cells was also detected. After being treated with TP solution, Exo/TP, and cRGD-Exo/TP (the concentration of TP was 60 ng/mL) for different times, A375 cells (living cells) were counted and lysed. The drug concentration in the lytic cells was analyzed using UHPLC-MS/MS.

#### Internalization mechanisms of cRGD-Exo/TP

A375 cells were pretreated with various endocytic inhibitors or cultured at low temperature to probe the internalization mechanisms of cRGD-Exo/TP [45]. A375 cells incubated in 12-well plates were pretreated with serum-free DMEM containing sodium azide (5 mg/mL), nystatin (50 μg/mL), chlorpromazine (5 μg/mL) and amiloride (50 μg/mL) for 1 h each. For the low-temperature group, the cells were treated with serum-free DMEM at 4 ℃. Then, the cells were washed with PBS and treated with 100 nM PHK67-labeled cRGD-Exo/TP for another 6 h at 37 °C. The cells cultured only with cRGD-Exo/TP at 37 ℃ were used as controls. The MFI of the PKH67 signal in different groups of cells was detected with flow cytometry.

#### Cytotoxicity assay

A375 cells were incubated with different concentrations of TP for 24 h. After that, 10 μL of CCK-8 was added to the treated cells and incubated for another 20 min. The absorbance was detected using a microplate reader. After that, the cytotoxicity of Exo, cRGD-Exo, TP solution, Exo/TP, and cRGD-Exo/TP was also evaluated (the concentration of TP in TP solution, Exo/TP, cRGD-Exo/TP was consistent with the IC50 of TP on cells, and the protein concentration of Exo was the same as TP).

#### Invasion assays

A375 cells were pretreated with Exo, cRGD-Exo, TP solution, Exo/TP, and cRGD-Exo/TP for 6 h and then trypsinized and resuspended in DMEM. Then, the differently treated cells were incubated in the apical Transwell insert (24-well plant covered with Matrigel polycarbonate membranes, pore size 8 μm). Meanwhile, 800 μL of DMEM with 10% FBS used for cell chemotaxis was added to the basolateral champers. After incubation for 24 h, the cells on the top side of the apical chamber were wiped, and then the migrated cells were fixed with 4% paraformaldehyde and stained with 0.1% crystal violet. After that, five random fields under an inverted microscope were visualized, and the migrated cells were counted by ImageJ software.

#### Cell apoptosis assay

To assess the proapoptotic effect of cRGD-Exo/TP on A375 cells, the cells were incubated with Exo, cRGD-Exo, TP solution, Exo/TP, and cRGD-Exo/TP for 24 h. Then, the cells were trypsinized by pancreatic enzymes without DMSO and resuspended in 500 μL 1× Binding Buffer. The cell suspension was stationarily cultured at room temperature for 15 min in the dark after adding 7-AAD and Annexin V-APC. Subsequently, cell apoptosis and necrosis were detected with a flow cytometer.

#### Cell cycle assay

The cell cycle assay was carried out to explore the correlation between mitosis and cytotoxicity. Briefly, A375 cells were incubated with DMEM, Exo, cRGD-Exo, TP solution, Exo/TP, and cRGD-Exo/TP for 24 h. The treated cells were then fixed with 70% ethanol for 12 h, washed with PBS 3 times, and stained with propidium iodide/RNase A for 30 min. After that, the red fluorescence of differently treated cells was recorded at 488 nm, and the DNA content of cells was also analyzed.

#### Western blotting analysis of apoptosis-related proteins

Western blotting was applied to investigate the mechanisms of cell apoptosis. The apoptosis-related proteins (caspase 3, caspase 8, caspase 9, Bcl-2, Bax, cyto *c*, NF-κB, VEGF, and survivin) of A375 cells incubated with DMEM and cRGD-Exo/TP (70 ng/mL TP) were detected by western blotting as described above.

#### In vivo biodistribution and pharmacokinetics of cRGD-Exo/TP

The xenograft tumor model was established by subcutaneously injecting 2–4 × 10^6^ A375 cells into the right flank of male BALB/c nude mice. When the tumor volume reached approximately 500 mm^3^, the mice were randomized into 2 groups (*n* = 3) and injected intravenously with 0.2 mL of DiR-labeled Exo/TP or DiR-labeled cRGD-Exo/TP (600 μg/kg of TP). After injection, mice were scanned at interval times (1, 2, 4, 6, 12, and 24 h) using an IVIS (Perkin-Elmer, USA) at 750/780 nm. Finally, mice were euthanized and surgically dissected at 24 h. The fluorescence intensity in the heart, liver, spleen, lung, kidney, blood, and tumors was also recorded.

The TP concentrations in blood, heart, liver, spleen, lung, kidney, and tumor tissues were also quantified over time. Briefly, 63 tumor-bearing nude mice were randomly divided into 3 groups (*n* = 21): TP solution, Exo/TP, and cRGD-Exo/TP. The preparations were injected intravenously at drug-equivalent doses of 600 μg/kg TP. After injection, blood was sampled from the orbital vein at 5 min, 10 min, 15 min, 30 min, 1 h, 2 h, 4 h, 6 h, and 24 h. In addition, heart, liver, spleen, lung, kidney, and tumor tissues were collected at 5 min, 30 min, 1 h, 2 h, 4 h, 6 h, and 24 h. Nine mice were sacrificed at each time point (*n* = 3), whereas at 10 min and 15 min, the mice were only taken for blood and were not killed. The TP concentration in plasma and tissue homogenate was analyzed with UHPLC-MS/MS.

#### In vivo* antitumor efficacy*

The xenograft tumor model mice were randomized into 5 groups (*n* = 6): (1) normal saline, (2) cRGD-Exo, (3) TP solution, (4) Exo/TP, and (5) cRGD-Exo/TP. The dosage of TP in the TP solution, Exo/TP, and cRGD-Exo/TP groups was 600 μg/kg, while that of Exo in the cRGD-Exo, Exo/TP, and cRGD-Exo/TPgroups was also 600 μg/kg. The first day was recorded as 0 d. Before each dose, the length (L) and width (W) of the tumors were measured to calculate tumor volume (TV, TV = L × W^2^/2). After 8 doses (dosing every other day for 14 days), the mice were sacrificed, and then the tumor tissues were acquired, weighed, photographed, and stained for H&E, TUNEL assay, and Ki67 staining. The tumor inhibition rate was calculated using Eq. (4). In addition, the survivorship curves were also profiled according to the survival time of each group of mice (*n* = 6).

4 $${\text{Tumor inhibition rate}}\, = \,\left( {{\text{Wc}}\, - \,{\text{Wa}}} \right)/{\text{Wc}}\, \times \,{1}00\%$$

where, Wc and Wa indicate the average weight of tumors in the control and administration groups, respectively.

#### Biosafety

The biosafety of cRGD-Exo/TP was also assessed based on the “in vivo antitumor efficacy” experiment. Briefly, the mouse bodyweight of all groups was recorded throughout the treatment period. To evaluate systemic safety, after the termination of treatment, the tissues of the heart, liver, spleen, lung, and kidney were collected to calculate organ indexes [mass of the organ (mg)/bodyweight (g)] and to stain for H&E. In addition, orbital blood was sampled for a routine blood test, hepatic function test and renal function test, including RBC, WBC, ALT, and Cr.

#### UHPLC-MS/MS analysis

TP concentrations in cells, plasma, primary organs, and tumors were analyzed using UHPLC-MS/MS (Thermo Fisher Scientific, CA, USA). The separation was performed on an ACQUITY UPLCTM BEH C18 column (2.1 × 100 mm, i.d., 1.7 μm). The column temperature was maintained at 40 °C. The mobile phases were 1% acetic acid aqueous solution (A) and methanol (B). Isocratic elution with 40% A and 60% B was adopted at a flow rate of 0.3 mL/mL. The temperature and flow rate of dry gas (N_2_) were 350 °C and 8 L/min, respectively. The pressure of the nebulizer gas (N_2_) was 40 psi. The capillary voltage was 4000 eV. The positive multiple reaction monitoring (MRM) mode was adopted to determine TP and carbamazepine (internal standard, IS). The collision energy was 33 eV for both TP and IS. The precursor-to-product transitions were m/z 361.1 → 105.2 for TP and m/z 237.1 → 194.0 for IS.

#### Statistical analysis

The results are presented as the mean of at least three experiments with the corresponding standard deviation (SD). Statistical data were analyzed using SPSS software, and a statistically significant difference was denoted by the difference probability level (p < 0.05). The t-test and one-way ANOVA were used to analyze the statistical data.

## Supplementary Information

**Additional file 1:** **Fig. S1**. Morphology of human umbilical cord mesenchymal stem cells. (A) Primary cells (bar: 100 μm). (B) Passage 3 cells (bar: 200 μm). **Fig. S2**. Zeta potential of Exo (A), cRGD-Exo (B), and cRGD-Exo/TP (C). **Fig. S3**. Size change of cRGD-Exo/TP stored at 4°C in PBS for 7 days. **Fig. S4.** Size change of cRGD-Exo/TP stored at 37°C in 10% Exo-free serum for 24 h. **Fig. S5.** Viability of A375 cells treated with TP solution at concentrations of 10, 20, 40, 60, 80, and 100 ng/mL. **Fig. S6.** αvβ3 integrin protein expression in A375 and HaCaT cells. (A) Western blotting analysis of αvβ3 protein expression in A375 and HaCaT cells. (B) The relative expression levels of the protein in the cells. GAPHD was used as a loading control. **Fig. S7.** TP concentration in the heart, liver, spleen, lung, and kidney of the TP solution, Exo/TP, and cRGD-Exo/TP groups at 0.083, 0.5, 1, 2, 4, 6, and 24 h.

## Data Availability

All data generated or analyzed during this study are included in this article.

## Abbreviations

TP: Triptolide
Exo: Exosome
hUCMSCs: Human umbilical cord mesenchymal stromal
cRGD: Cyclic arginine-glycine-aspartate peptide
MM: Malignant melanoma
MPS: Mononuclear phagocyte system
MSCs: Mesenchymal stromal cells
TEM: Transmission electron microscopy
DLS: Dynamic light scattering
PDI: Polydispersity index
HRP: Horseradish peroxidase-conjugated
CLSM: Confocal laser scanning microscope
MFI: Mean fluorescence intensity
IC50: Half-maximal inhibitory concentration
IVIS: *in vivo* Imaging system
IS: Internal standard
H&E: Histopathological examination
RBC: Red blood cell
WBC: White blood cell
ALT: Alanine aminotransferase
Cr: Creatinine
SD: Standard deviation
