# Supplementary material for: αvβ3 integrin-specific exosomes engineered with cyclopeptide for targeted delivery of triptolide against malignant melanoma
Source: J Nanobiotechnology. 2022 Aug 23;20:384. doi: 10.1186/s12951-022-01597-1 (PMC9400227; doi:10.1186/s12951-022-01597-1)
Supplement: Supplementary file 1 — Additional file 1: Fig. S1. Morphology of human umbilical cord mesenchymal stem cells. (A) Primary cells (bar: 100 μm). (B) Passage 3 cells (bar: 200 μm). Fig. S2. Zeta potential of Exo (A), cRGD-Exo (B), and cRGD-Exo/TP (C). Fig. S3. Size change of cRGD-Exo/TP stored at 4°C in PBS for 7 days. Fig. S4. Size change of cRGD-Exo/TP stored at 37°C in 10% Exo-free serum for 24 h. Fig. S5. Viability of A375 cells treated with TP solution at concentrations of 10, 20, 40, 60, 80, and 100 ng/mL. Fig. S6. αvβ3 integrin protein expression in A375 and HaCaT cells. (A) Western blotting analysis of αvβ3 protein expression in A375 and HaCaT cells. (B) The relative expression levels of the protein in the cells. GAPHD was used as a loading control. Fig. S7. TP concentration in the heart, liver, spleen, lung, and kidney of the TP solution, Exo/TP, and cRGD-Exo/TP groups at 0.083, 0.5, 1, 2, 4, 6, and 24 h. [file 12951_2022_1597_MOESM1_ESM.docx]

αvβ3 integrin-specific exosomes engineered with cyclopeptide for targeted delivery of triptolide against malignant melanoma

Yongwei Gu^1†^, Yue Du^1,2†^, Liangdi Jiang^1,3†^, Xiaomeng Tang^1^, Aixue Li^1^, Yunan Zhao^1^, Yitian Lang^4^, Xiaoyan Liu^4,5^*, Jiyong Liu^1^*

^1^Department of Pharmacy, Fudan University Shanghai Cancer Center; Department of Oncology, Shanghai Medical College, Fudan University, Shanghai 200032, China

^2^Department of Pharmacy, Children’s Hospital Affiliated to Shanghai Jiao Tong University, Shanghai 200062, China

^3^ School of Pharmacy, Shanghai Jiao Tong University, Shanghai 200240, China

^4^Department of Pharmacy, Huangpu Branch, Shanghai Ninth People's Hospital, Shanghai Jiao Tong University School of Medicine, Shanghai 200011, China.

^5^State Key Laboratory of Quality Research in Chinese Medicine & School of Pharmacy, Macau University of Science and Technology, Avenida Wai Long, Taipa, Macau SAR, 999078, China.

**^†^**These authors made equal contributions to this work.

*Corresponding author.

Jiyong Liu,

Tel/Fax: +86-21-64175590, E-mail address: [liujiyong@fudan.edu.cn](mailto:liujiyong@fudan.edu.cn)

Xiaoyan Liu,

Tel/Fax: +86-21-23308784, E-mail address: [liuxiaoyanrj@sjtu.edu.cn](mailto:liuxiaoyanrj@sjtu.edu.cn)


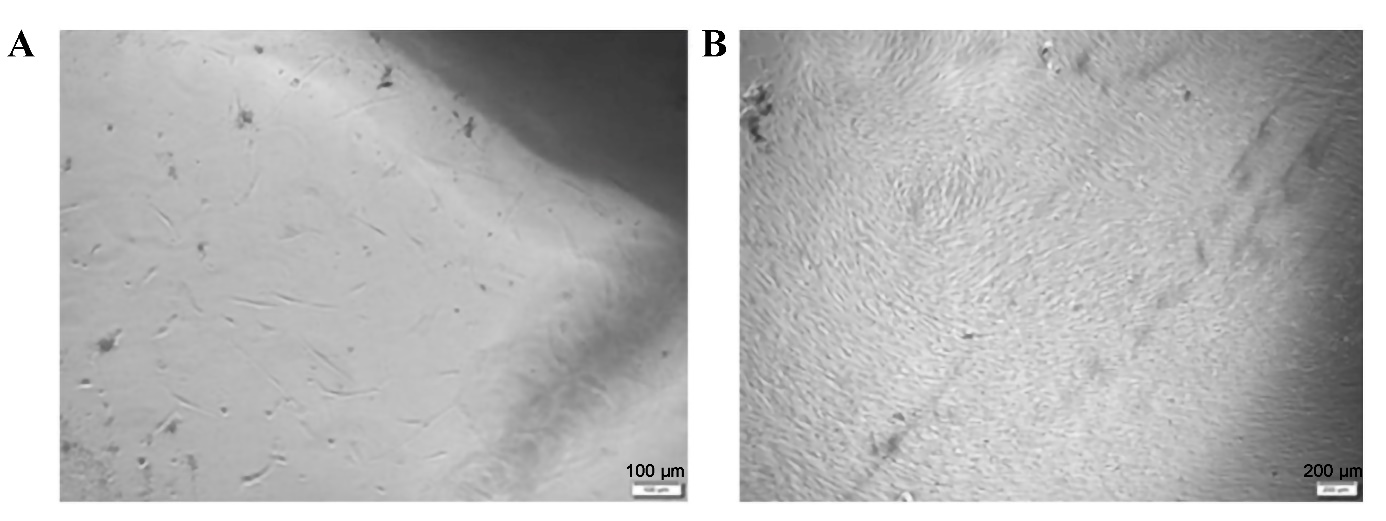


**Figure S1** The morphology of human umbilical cord mesenchymal stem cells. (A) primary cells (bar: 100 μm). (B) Passage 3 cells (bar: 200 μm).


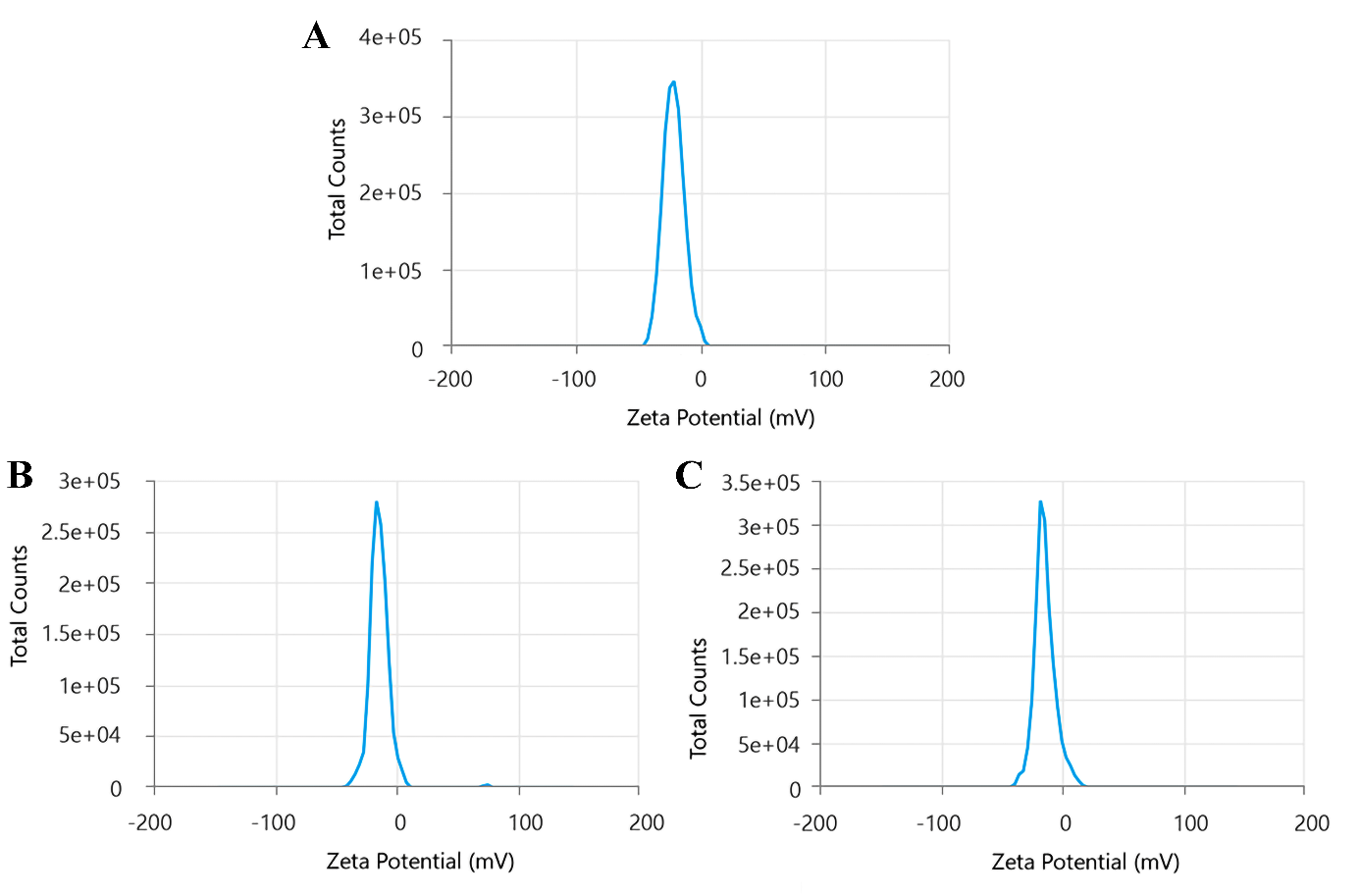


**Figure S2** Zeta potential of Exo (A), cRGD-Exo (B), and cRGD-Exo/TP (C).


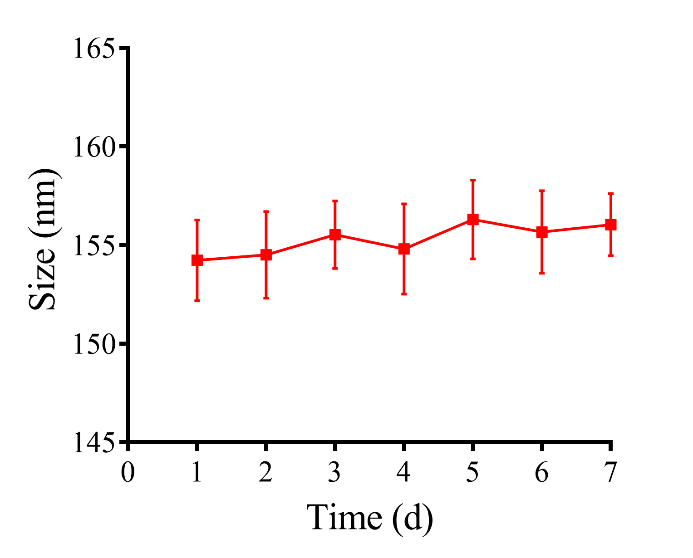


**Figure S3** Size change of cRGD-Exo/TP stored at 4°C in PBS for 7 days.


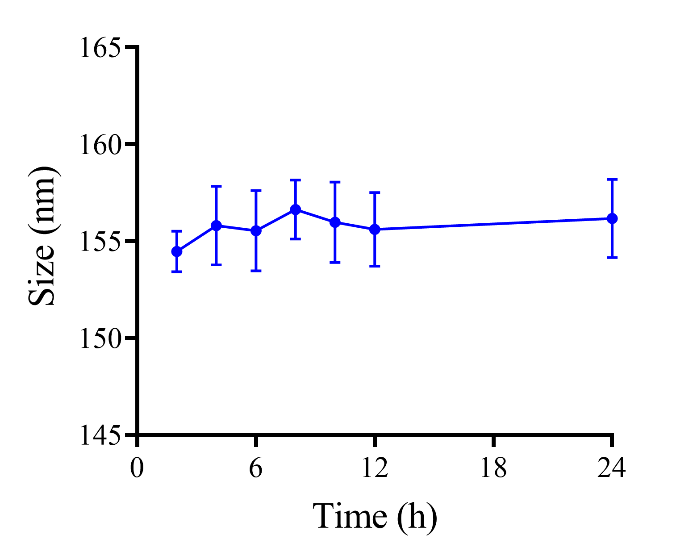


**Figure S4** Size change of cRGD-Exo/TP stored at 37°C in 10% Exo-free serum for 24 h.


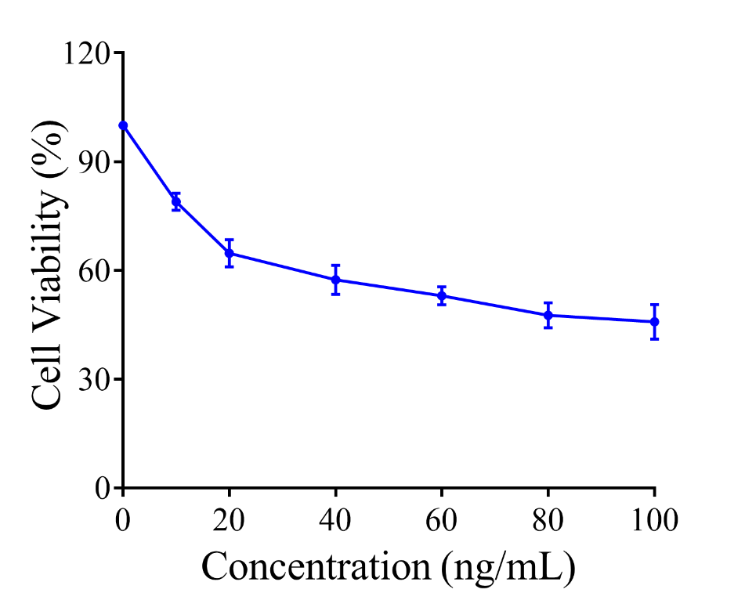


**Figure S5** Viability of A375 cells treated with TP solution with the concentration of 10, 20, 40, 60, 80, and 100 ng/mL.


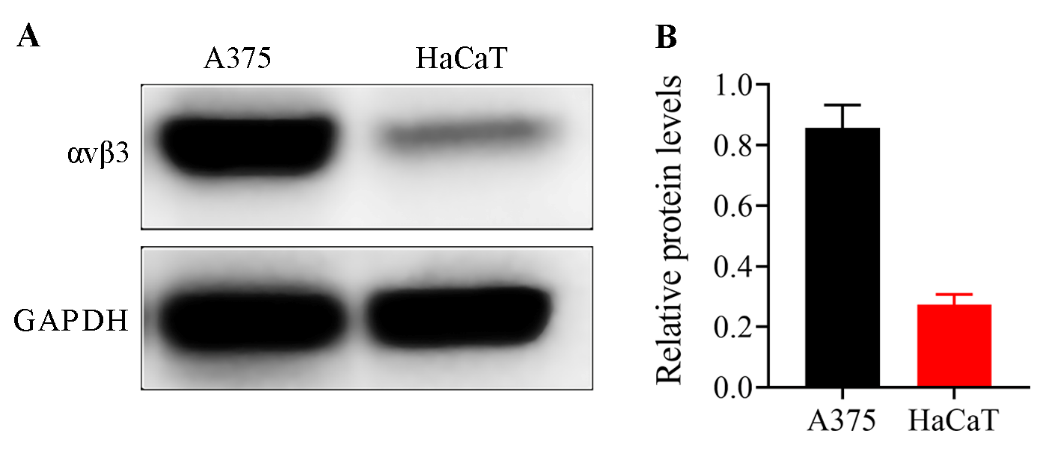


**Figure S6** αvβ3 integrin protein expression in A375 and HaCaT cells. (A) Western blotting analysis of αvβ3 protein expression in A375 and HaCaT cells. (B) The relative expression levels of the protein in the cells. GAPHD was used as a loading control.


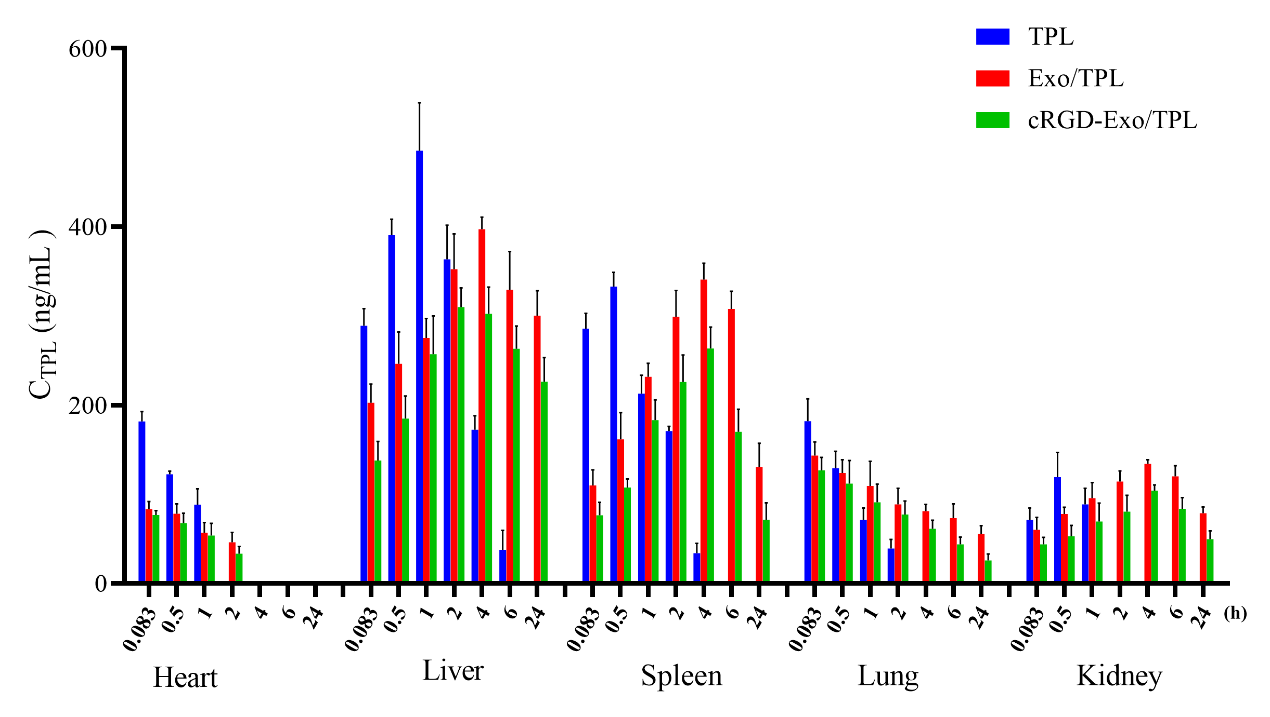


**Figure S7** TP concentration in heart, liver, spleen, lung, and kidney of TP solution, Exo/TP, and cRGD-Exo/TP groups at 0.083, 0.5, 1, 2, 4, 6, and 24 h.
